# The complete chloroplast genome of *Camellia nitidissima* (Theaceae)

**DOI:** 10.1080/23802359.2020.1768957

**Published:** 2020-05-28

**Authors:** Shangli Liu, Siyu Pei, Lin Huang, Shaoqing Tang

**Affiliations:** a Key Laboratory of Ecology of Rare and Endangered Species and Environmental Protection, Ministry of Education, Guangxi Normal University, Guilin, China; b Guangxi Key Laboratory of Rare and Endangered Animal Ecology, College of Life Science, Guangxi Normal University, Guilin, China

**Keywords:** *Camellia nitidissima* var. *nitidissima*, *Camellia nitidissima* var. *microcarpa*, chloroplast genome, phylogenetic relationships

## Abstract

*Camellia nitidissima* is an endangered species. This species contains two varieties. Here, we report on the chloroplast genomes of *C. nitidissima* var. *nitidissima* from Fangcheng (GenBank accession MT157617) and Nanning (MT157618), as well as one sample of *C*. *nitidissima* var. *microcarpa* (MT157619) from Nanning. The total chloroplast genomes of *C. nitidissima* var. *nitidissima* Fangcheng and Nanning samples are 156,596 bp and 157,567 bp in length, respectively. *C. nitidissima* var. *microcarpa* (MT157619) genome is 157,407 bp in length. The three samples possess GC contents of 37.3%, 128 genes, comprising 86 protein-coding genes, 34 tRNA genes, and 8 rRNA genes.

*Camellia nitidissima* Chi, belonging to family Theaceae, is distributed across southern China and northern Vietnam (Chang and Ren 1998). *Camellia nitidissima* contains two varieties, *C. nitidissima* var. *nitidisima* and *C. nitidissima* var. *microcarpa*. It is an ornamental plant and its flowers and leaves can be used as a tea. Because of its high economic value and lack of effective protection, *C. nitidissima* is seriously exploited*. C. nitidissima* var. *nitidissima* is classified as “vulnerable”, and *C. nitidissima* var. *microcarpa* as “endangered” (Qin et al. 2017). The complete chloroplast (cp) genome of *C. nitidissima* (GenBank accession NC039645) has been published (Liu et al. 2018); however, this study did not consider the disjunctive geographical distribution of *C. nitidissima* var. *nitidissima* in Guangxi, China, or high genetic variation of cpDNA sequences from the two disjunctive areas (Lu et al. 2020). Here, we present cp genome sequences of *C. nitidissima* var. *nitidissima* from Fangcheng (GenBank accession MT157617) and Nanning (MT157618), as well as the cp genome sequence of one *Camellia nitidissima* var. *microcarpa* sample (MT157619) from Nanning.

*Camellia nitidissima* var*. nitidissima* samples were collected from two disjunctive distribution areas, Nanning Golden Camellia Park and Fangcheng Yellow Camellia National Nature Reserve in Guangxi, China, while *C. nitidissima* var*. microcarpa* was collected from Nanning. The Voucher specimens (Liushangli20191215, Liushangli20191216, Liushangli20191231) were deposited in the herbarium of Guangxi Institute of Botany (IBK). GenBank accession numbers for *C. nitidissima* var. *natidisisma* are MT157617 and MT157618, and the GenBank accession number for *C. nitidissima* var. *microcarpa* is MT157619. Total genomic DNA was extracted from fresh leaves by using the Super Plant Genomic DNA Kit (TIANGEN, Beijing, China). The cp genome was sequenced using an Illumina HiSeq 400 platform. The Filtered reads were assembled with NOVOPlasty (https://github.com/ndierckx/NOVOPlasty). Annotation was performed with the Plastid Genome Annotator (PGA, https://github.com/quxiaojian/PGA).

The cp genome of *C. nitidisima* var. *nitidissima* (MT157617) collected from Fangcheng is 156,596 bp in length with 37.3% GC contents, and, consist of a large single-copy (LSC) region (86,272 bp), a small single-copy (SSC) region (18,252 bp), separated by a pair of inverted repeat (IRs: 26,036 bp, each) regions. There are 86 protein-coding genes, 8 rRNA genes, and 34 tRNA genes. The cp genome of *C. nitidissima* var. *nitidissima* (MT157618) collected from Nanning is 157,567 bp in length with 37.3% GC contents, and consist of a LSC region (86,632 bp), and, a SSC region separated (17,735 bp) separated by a pair of IRs (26,600 bp) regions. There are 86 protein-coding genes, 8 rRNA genes, and 34 tRNA genes. The cp genome of *C. nitidissima* var. *microcarpa* (MT157619) collected from Nanning is 157,047 bp in length with 37.3% GC content, and consist of an LSC region (86,626 bp), an SSC region (18,277 bp), and a pair of IRs: 26,072 bp, each. There are 86 protein-coding genes, 8 rRNA genes, and 34 tRNA genes.

We reconstructed a phylogenetic tree, which was based on 22 complete chloroplast genome sequences, including those of *C. nitidissima* var. *nitidissima* (MT157617, MT157618) and *C. nitidissima* var. *microcarpa* (MT157619), 18 Camellia species, and the outgroup *Polyspora axillaris*. Genome sequences were aligned using the software Mafft (Katoh and Standley 2013), and the phylogenetic tree was generated by the software RaxML (Stamatakis 2014) using maximum-likelihood method with 1000 bootstrap replicates (Figure 1). The tree shows that *C. nitidissima* var. *nitidissima* (MT157618) from Nanning was closely related to *C. nitidissima* var. *microcarpa* (MT157619) distributed from Nanning than to the *C. nitidissima* var. *nitidissima* sample (MT157619) from Fangcheng (Figure 1). The complete chloroplast genome of *C. nitidissima* provides essential data for the conservation and utilization of this species.

**Figure 1.**
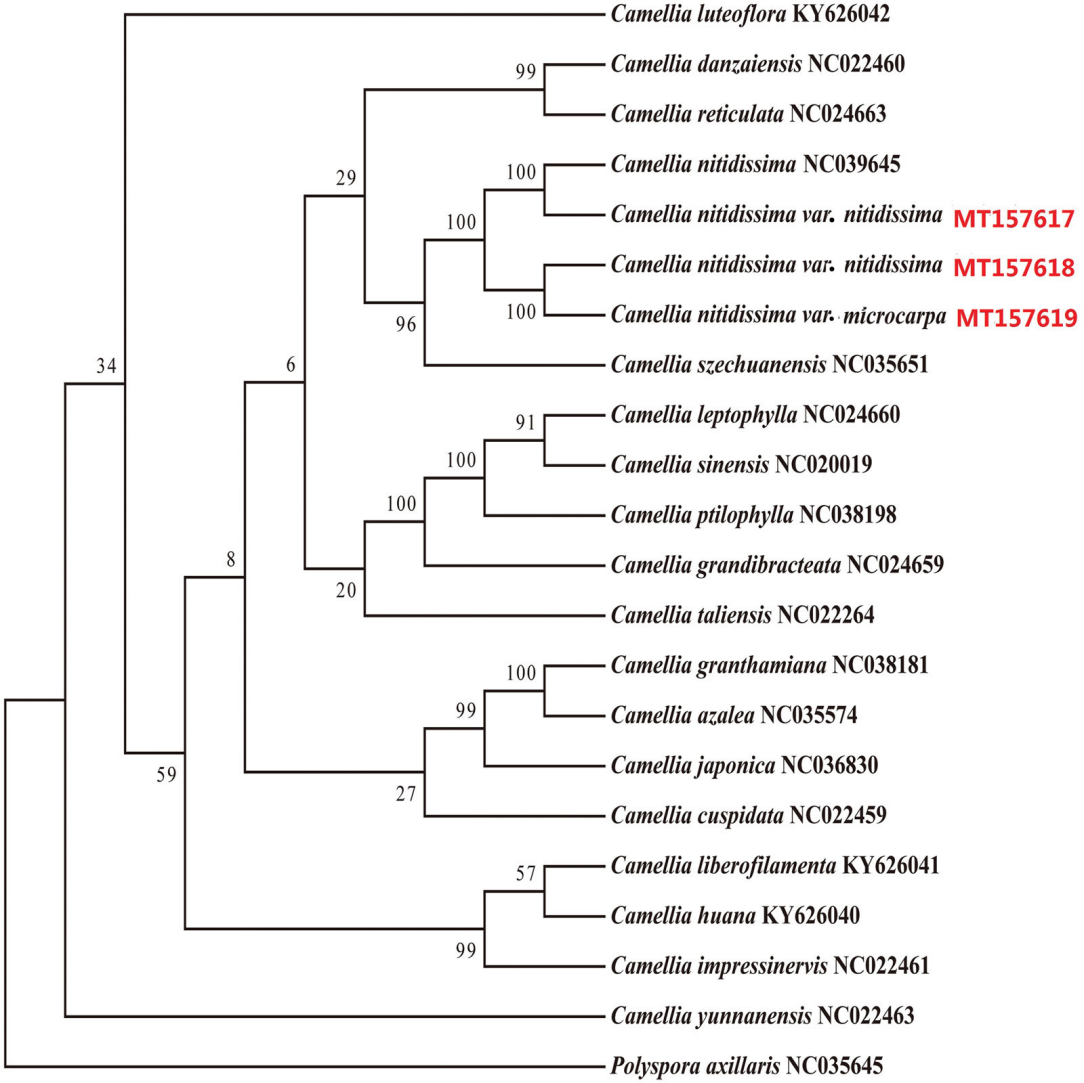
The phylogenetic tree was constructed based on 22 chloroplast genome sequences using the maximum-likelihood method.

## Data Availability

The data that support the findings of this study are available in GenBank, reference number (MT157617, MT157618,MT157619). These data were derived from the following resources available in the public domain: [constructed phylogenetic tree: NC035574, NC038181, NC036830, NC022459, NC022264, NC020019, NC024660, NC038198, NC024659, NC024663, NC022460, NC035651, NC039645, NC022461, KY626040, KY626041, KY626042, NC022463, NC035645, https://www.ncbi.nlm.nih.gov/genbank/]
